# Striving for consistency in the National Wetland Condition Assessment: developing a reference condition approach for assessing wetlands at a continental scale

**DOI:** 10.1007/s10661-019-7325-3

**Published:** 2019-06-20

**Authors:** Alan T. Herlihy, Mary E. Kentula, Teresa K. Magee, Gregg A. Lomnicky, Amanda M. Nahlik, Gregg Serenbetz

**Affiliations:** 1 Department of Fisheries & Wildlife, Oregon State University, 104 Nash Hall, Corvallis, OR 97331 USA; 2 Present Address: National Health and Environmental Effects Research Laboratory - Western Ecology Division, US Environmental Protection Agency, 200 SW 35th St., Corvallis, OR 97333 USA; 3 CSS-Dynamac Corporation, 200 SW 35th St., Corvallis, OR 97333 USA; 4 Department of Biology, Kenyon College, 202 N. College Road, Gambier, OH 43022 USA; 5 Office of Water, US Environmental Protection Agency, 1200 Pennsylvania Ave., NW, MC4502T, Washington DC, 20460 USA

**Keywords:** Wetlands, Reference condition, Reference sites, Regionalization, Regional assessments

## Abstract

One of the biggest challenges when conducting a continental-scale assessment of wetlands is setting appropriate expectations for the assessed sites. The challenge occurs for two reasons: (1) tremendous natural environmental heterogeneity exists within a continental landscape and (2) reference sites vary in quality both across and within major regions of the continent. We describe the process used to set reference expectations and define a disturbance gradient for the United States (US) Environmental Protection Agency’s National Wetland Condition Assessment (NWCA). The NWCA employed a probability design and sampled 1138 wetland sites across the conterminous US to make an unbiased assessment of wetland condition. NWCA vegetation data were used to define 10 reporting groups based on ecoregion and wetland type that reduced the naturally occurring variation in wetland vegetation associated with continent-wide differences in biogeography. These reporting groups were used as a basis for defining quantitative criteria for least disturbed and most disturbed conditions and developing indices and thresholds for categories of ecological condition and disturbance. The NWCA vegetation assessment was based on a reference site approach, in which the least disturbed reference sites were used to establish benchmarks for assessing the condition of vegetation at other sites. Reference sites for each reporting group were identified by filtering NWCA sample data for disturbance using a series of abiotic variables. Ultimately, 277 least disturbed sites were used to set reference expectations for the NWCA. The NWCA provided a unique opportunity to improve our conceptual and technical understanding of how to best apply a reference condition approach to assessing wetlands across the US. These results will enhance the technical quality of future national assessments.

## Introduction

The primary goals of the United States Environmental Protection Agency’s (USEPA) National Wetland Condition Assessment (NWCA) were to evaluate the ecological condition of wetlands in the United States (US) and rank the stressors that might affect them. The ecological condition indicator for the NWCA was a multimetric index of vegetation condition (VMMI; Magee et al. 2019a). This assessment technique relies on a reference condition approach (Bailey et al. 2004; Hawkins et al. 2010) to set expectations for vegetation in individual wetlands. Reference expectations should be class- or even site-specific because of natural variation in environmental conditions. Reference condition for an assessed site is inferred from information collected at reference sites within a region and/or wetland type. Reference sites are also used in wetland assessments to develop reference standard condition for assessing wetland function using the hydrogeomorphic (HGM) approach (Brinson 1993; Brinson and Rheinhardt 1996; Rheinhardt et al. 1997). Reference sites provide the benchmark against which the ecological conditions of all other wetlands in a class are measured. In the NWCA, reference sites were used for two purposes: (1) to develop and calibrate the VMMI assessment model and (2) to set the thresholds used to divide continuous assessment variables into good, fair, or poor condition classes.

Identification of reference sites is difficult and time-consuming. Ideal reference sites would be unaffected by human activities. However, it is doubtful that any such sites exist in the conterminous US as every site is impacted to some degree by atmospheric deposition of pollutants. Sometimes locations can be identified that have experienced a minimal degree of human influence (e.g., wilderness areas). Sites in these areas generally are thought to be in a minimally disturbed reference condition (Stoddard et al. 2006). However, these locations are also rare and often provide a comparison for only a specific subset of wetlands. In most other cases, least disturbed reference sites (Stoddard et al. 2006) must be used, and assessments are done by comparing sites to be assessed with the highest quality sites remaining within the study area. The selection of least disturbed sites is usually based on either best professional judgment or by screening abiotic data for human disturbance indicators (Herlihy et al. 2008; Whittier et al. 2007).

For the NWCA, the problem of identifying appropriate and comparable reference sites was greatly complicated by the continental scale of the assessment. US wetlands are extremely heterogeneous with respect to many natural environmental attributes. Thus, reference sites had to be selected to characterize this range of natural heterogeneity. One of our challenges was to develop a classification scheme that would minimize the effect of natural environmental variation on indicator values while providing large enough sample sizes to allow statistically valid assessments within ecologically meaningful subpopulations of the US. Furthermore, the degree of landscape alteration that has occurred in different parts of the US varies greatly, so the availability and overall quality of reference sites also varies among regions and types of wetlands.

We describe here our efforts to define consistent reference conditions for the NWCA. First, we present our scheme for partitioning the effects of natural environmental heterogeneity on vegetation structure and composition by developing reporting groups. These reporting groups were used in the NWCA to develop group-specific: (1) quantitative criteria for least disturbed and most disturbed condition, (2) indicators and indices for reporting on ecological condition and stressor extent, and (3) thresholds for categories of ecological condition and disturbance. Next, we discuss our approach for screening and assembling a reference site data set large enough to characterize the condition of wetlands in the conterminous US. Lastly, we define a disturbance gradient for NWCA sites based on classifying sites into least, intermediate, and most disturbed groups.

## Methods

### NWCA design and site selection

The purpose of the USEPA’s National Aquatic Resource Surveys (NARS) is to generate statistically valid and environmentally relevant reports on the condition of the nation’s aquatic resources every 5 years. The NWCA is one of the NARS along with national surveys of lakes, streams, rivers, and near coastal systems. The goals of the NWCA are to (1) produce a report describing the ecological condition of the nation’s wetlands and anthropogenic stressors commonly associated with poor condition; (2) collaborate with states and tribes in developing complementary monitoring tools, analytical approaches, and data management technology to aid wetland protection and restoration programs; and (3) advance the science of wetland monitoring and assessment to support wetland management needs (see USEPA 2016a).

The NWCA was designed to assess the regional ecological condition of broad groups or populations of wetlands, rather than individual wetlands or wetlands within individual states or watersheds. The NWCA design allows characterization of wetlands at national and regional scales using indicators of ecological condition and stress. The target population for the NWCA was all wetlands of the conterminous US not currently in crop production, including tidal and nontidal wetted areas with rooted vegetation and, when present, shallow open water less than 1 m in depth (Olsen et al. 2019). A wetland’s jurisdictional status under state or federal regulatory programs did not factor into this definition. Wetland attributes are assumed to vary continuously across a wetland.

The selection of the probability sites was completed in two steps as described by Olsen et al. (2019). Since a consistent national digital map of all wetlands in the conterminous US was not available to draw the sample and the US Fish & Wildlife Service conducts the National Wetland Status and Trends (S&T) survey, the approximately 5000 4-mi^2^ (10.4 km^2^) plots based on 2005 photography from S&T were used to identify wetlands in the NWCA target population in the first step. In the second step, a Generalized Random Tessellation Stratified survey design (Stevens Jr. and Olsen 1999, 2004) for an area resource was applied to the S&T wetland polygons. This step was stratified by state with unequal probability of selection by NWCA wetland type.

Probability sites from the NWCA survey design were screened using aerial photo interpretations and GIS analyses to eliminate locations not suitable for NWCA sampling (e.g., non-NWCA wetland types, wetlands converted to non-wetland land cover due to development). Sites could also be eliminated during field reconnaissance if, for example, they were a non-target type or could not be assessed due to accessibility or safety issues. Dropped sites were systematically replaced from a pool of replacement sites from the random design. Details of the NWCA sampling design and site selection are described in the NWCA 2011 Technical Report (USEPA 2016b) and Olsen et al. (2019). The NWCA survey design and resulting sampled probability sites allow estimation of wetland area in different condition categories (good, fair, poor) across the conterminous US. These extent estimates are described in the 2011 NWCA Final Report (USEPA 2016a).

**Fig. 1:**
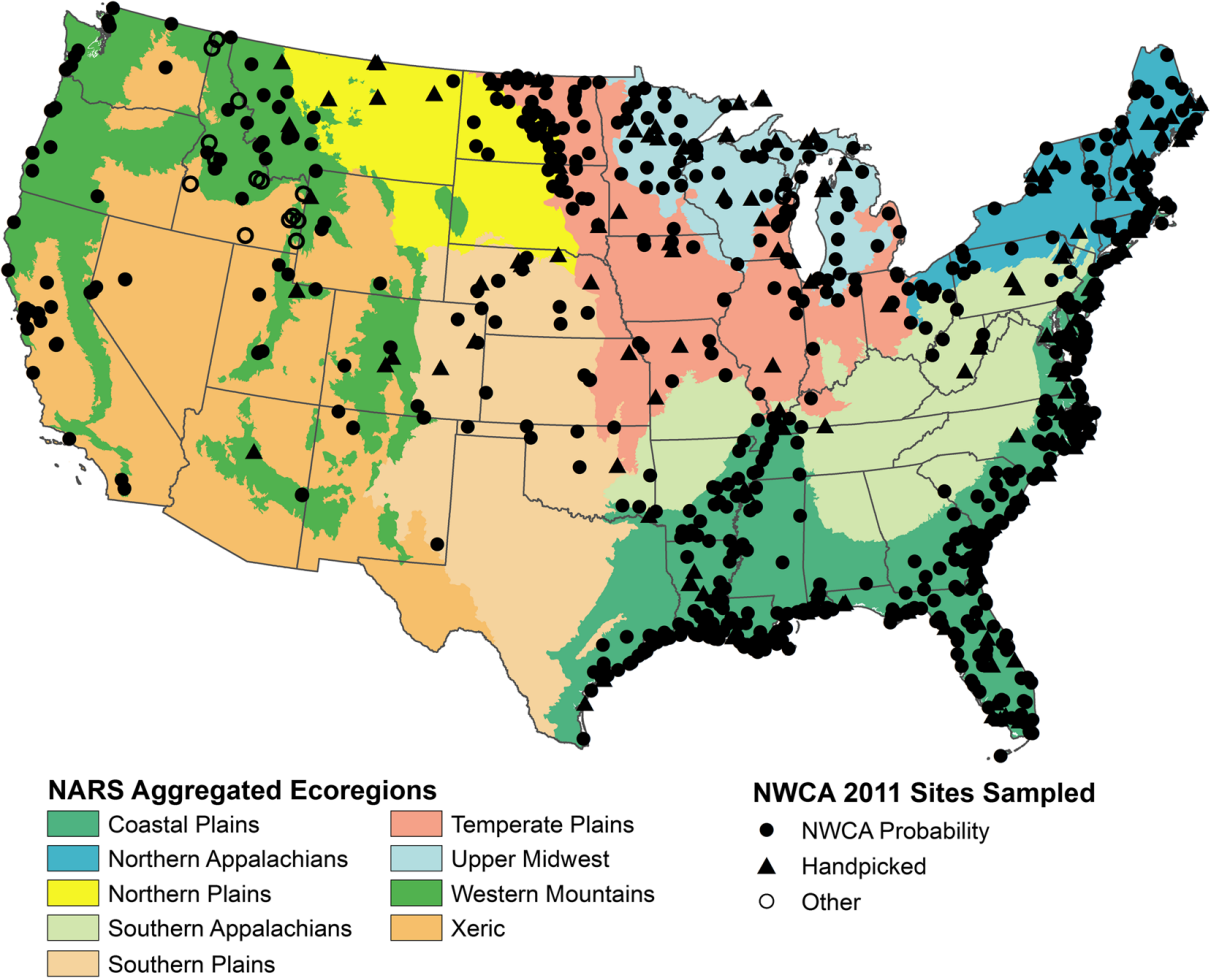
Map of the conterminous US showing distribution of handpicked sites in relation to NWCA probability sites and other sites sampled in the 2011 NWCA. The nine aggregated ecoregions are combinations of level III ecoregions (Omernik 1987) and are used in other NARS assessments (Herlihy et al. 2008)

A total of 967 probability sites from the NWCA survey design were sampled. The spatial distribution of these probability sites was not uniform (Fig. 1), but reflects the distribution of wetlands in the nation based on the S&T sample frame. For example, wetlands are more common in coastal areas of the US, particularly in the Southeast. In addition to the NWCA probability sites, another 171 sites were sampled. To augment the number of potential reference sites, 150 handpicked sites thought to be in good condition were chosen for sampling. Lastly, 21 additional sites were sampled as part of the NWCA for various state-level studies and we have just labeled these as “other sites.” All 1138 sampled sites (Fig. 1, Table 1) were used in the analyses in this paper and each site was evaluated using quantitative screening criteria to determine its disturbance status as least (reference), intermediate, or most disturbed.

**Table 1:** Distribution of the number of NWCA probability sites, other sites, and handpicked sites by the nine NARS aggregated ecoregions (Fig. 1) and by the seven NWCA wetland types

	Number of NWCA probability sites	Number of other sites	Number of handpicked sites	Total number of sites
NARS aggregated ecoregion				
Coastal Plain (CPL)	513	0	54	567
Southern Appalachians (SAP)	22	0	7	29
Northern Appalachians (NAP)	80	0	29	109
Upper Midwest (UMW)	50	2	24	76
Temperate Plains (TPL)	94	5	14	113
Northern Plains (NPL)	29	0	8	37
Southern Plains (SPL)	33	0	7	40
Xeric West (XER)	59	3	0	62
Western Mountains (WMT)	87	11	7	105
Total NWCA	967	21	150	1138
NWCA wetland type				
Estuarine emergent (EH) [estuarine herbaceous]	258	0	14	272
Estuarine shrub/forest (EW) [estuarine woody]	69	0	4	73
PRL-emergent (PRL-EM)	262	5	43	310
PRL-unconsolidated bottom/aquatic bed (PRL-UBAB)	18	0	8	26
PRL-farmed (PRL-f); subset not actively farmed	22	0	0	22
PRL-shrub/scrub (PRL-SS))	115	8	31	154
PRL-forested (PRL-FO)	223	8	50	281
Total NWCA	967	21	150	1138

*PRL* palustrine, shallow riverine, or shallow lacustrine

Selection of the 150 handpicked sites is described in USEPA (2016b). In brief, candidate handpicked sites originated from three sources: (1) best professional judgment (BPJ) recommendations from collaborators, (2) partner organizations conducting wetland assessments, and (3) in-field replacements for handpicked sites that were not sampleable or were significantly disturbed. Initially, 1264 candidate BPJ sites were identified and screened to eliminate sites that fell outside of the target population, were too close to a probability site, or which had difficult or unsafe access. As field data were lacking for most candidate sites, sites underwent additional landscape screening to remove those likely to have excessive levels of human impact. The landscape screen involved quantitative scoring of land use or land cover and presence of roads, trails or ditches in a 1-km radius, circular buffer around the site using aerial photography. Based on this screening, a total of 107 potential least disturbed sites were selected for sampling after taking into account the goal of distributing sites across NWCA wetland types and ecoregions. An additional 43 sites from collaborator assessments were selected for sampling without landscape screening as they were presumed to have been vetted by collaborators. During 2011 sampling, some of the 150 handpicked sites were replaced with alternates based on field crew judgment, primarily due to access issues. Handpicked sites were sampled using the same field protocols and were evaluated as potential reference sites using the same criteria as for the NWCA probability and other sites.

### Data collection and development of disturbance indicators

All sites were sampled in 2011 during a sampling period that ranged from April to September depending on the growing season of the state in which the site was located. A 40-m radius, circular assessment area (AA) was defined around each sample point, either handpicked or randomly selected from the survey design (USEPA 2011a). If this was not possible, the shape and size of the AA could be adjusted to fit the site. Within the AA, field crews completed a human disturbance checklist (Lomnicky et al. 2019) and sampled soil (Nahlik et al. 2019) and vegetation (Magee et al. 2019a). When sufficient surface water was present, a water sample was also collected for analysis of chemistry (Trebitz et al. 2019). Field and laboratory methods for the NWCA are described in detail in USEPA (2011a, b). The development of site-level disturbance indicators is detailed in USEPA (2016b). An overview of the collection of disturbance data and description of the site-level disturbance indicators is presented here.

A human disturbance checklist was completed at 13 10 × 10-m plots at the site (Lomnicky et al. 2019). The first plot was at the center of the AA. The remaining 12 plots were laid out along the four cardinal directions (3 in each direction) in the buffer. For each transect, buffer plot 1 was at the edge of the AA (40 m from center); buffer plot 3 was placed at the farthest extent of the defined buffer usually 140 m from the AA center; and buffer plot 2, midway between plots 1 and 3. The human disturbance checklist data in the buffer were grouped into five categories: agriculture, residential/urban, industrial, hydrologic modification, and habitat modification (Table 2). A disturbance index was developed and calculated for each of these categories of buffer disturbance based on the proximity-weighted average of the number of human disturbances observed in each plot as described in Lomnicky et al. (2019). A value of 1 indicates that on average, one human disturbance of that disturbance category was observed in each of the plots at the site. The maximum value observed in the NWCA for any buffer disturbance category was 2.3, but it was rare for a site to have values greater than 1. An overall buffer disturbance index score was calculated for each site by summing the index scores for each of the five buffer disturbance categories (Table 2). In a similar fashion, the hydrologic disturbance checklist data were categorized into high and medium impact categories (Table 2). As the hydrologic disturbance data were only collected in the AA, there was no proximity-weighting and the index value was just the number of observed disturbances. The observed range in index values was 0–7 but values above 4 were very rare.

**Table 2:** Ten disturbance measures used to screen all sample sites and set the disturbance gradient

Index code	Disturbance	Disturbance index
B1H_AGR	Agriculture	Σ [pasture/hay, range, row crops, fallow field, nursery, dairy, orchard, CAFO, rural residential, gravel pit, irrigation] buffer stressors
B1H_RESURB	Residential and urban disturbance	Σ [road (gravel, two lane, four lane), parking lot/pavement, golf course, lawn/park, suburban residential, urban/multifamily, landfill, dumping, trash] buffer stressors
B1H_IND	Industrial disturbance	Σ [oil drilling, gas well, mine (surface, underground), military)]
B1H_HYD	Hydrologic modifications	Σ [ditches/channelization, dike/dam/road/railroad bed, water level control structure, excavation, fill, fresh sediment, soil loss/root exposure, wall/riprap, inlets, outlets, pipes (effluent/stormwater), impervious surface input (sheetflow)] buffer stressors
B1H_HAB	Habitat modifications	Σ [forest clear cut and selective cut, tree plantation, canopy herbivory, shrub layer browsed, highly grazed grasses, recently burned forest, recently burned grassland, herbicide use, mowing/ shrub cutting, trails, soil compaction, off road vehicle damage, soil erosion] buffer stressors
B1H_ALL	Summary	Σ [B1H_AGR, B1H_RESURB, B1H_IND, B1H_HYD, B1H_HAB]
HDIS_HIGH	High impact hydrologic disturbances	Σ [damming features (dikes, berms, dams, railroad bed, roads), impervious surfaces (road, concrete, asphalt), pumps, pipes, culverts, ditches, excavation, field tiling] hydrologic disturbances in AA
HDIS_MED	Moderate impact hydrologic disturbances	Σ [shallow channels (animal trampling, vehicle ruts), recent sedimentation] hydrologic disturbances in AA
HMI	Heavy metals	Of the 12 heavy metals assessed (Ag, Cd, Co, Cr, Cu, Ni, Pb, Sb, Sn, V, W, Zn), the number of metals that had surface soil concentrations above background. The index value can range from 0 to 12
ALIENSPP	Alien species	% alien vegetation species cover

*CAFO* combined animal feeding operations, *AA* assessment area

Four soil pits were dug to a depth of 60 cm and one soil pit was chosen as the representative pit and expanded to 125 cm deep (USEPA 2011a). At the representative pit, soil chemistry samples were collected from each soil layer greater than 8 cm thick and sent to the lab for an extensive chemical analysis (Nahlik et al. 2019). In screening for least disturbed sites, we focused only on heavy metal concentration data from the uppermost layer collected and analyzed from each site. Almost all sites (97%) with soils data had chemistry from a layer that began within 10 cm of the surface. In the laboratory, metals were measured by inductively coupled plasma atomic emission spectrophotometry. A heavy metal disturbance index (HMI) was calculated for each site based on the concentrations of 12 different heavy metals (Table 2). Nahlik et al. (2019) derived a background concentration for each metal and the HMI is simply the number of metals at each site that exceeded their background concentration. Thus, the HMI can range from 0 to 12. About 10% of the sites had no soil data due to difficulties in obtaining samples.

For vegetation, five 100-m^2^ plots were systematically laid out in the AA. All plant species in the plots were identified to species, and estimates of abundance for each species (absolute percent cover) were also made. All sites were successfully sampled for vegetation. Plant species that were alien (introduced or adventive) for each location (Magee et al. 2019b) were quantified and a metric describing the relative percent cover of alien plant species (hereafter, alien plant cover) was calculated for each site (USEPA 2016b).

We used available GIS data layers to develop landscape disturbance indicators to evaluate our categorization of reference condition. The landscape indicators described land use and land cover for agriculture and combined urban/residential/industrial development and were based on the 2006 National Land Cover Database (NLCD, Homer et al. 2007). The percent area with disturbances from agriculture (planted/cultivated classes 81 and 82) and development (developed classes 21–24) were calculated within a circular buffer with a 1-km radius around the sample point using ArcGIS.

### Developing reporting groups

Appropriate expectations for reference condition must be adjusted for the natural conditions at a site (Herlihy et al. 2008). One approach to account for natural variation specifies site-specific reference condition using modeling of continuous environmental gradients to predict expected reference site biota (e.g., Moss et al. 1987) or to predict the values for multimetric indices of biological condition that would be expected under reference conditions (Cao et al. 2007; Pont et al. 2009). However, a more widely used, and perhaps more intuitively interpretable, approach for making adjustments for natural conditions is classification (or regionalization). A regional classification scheme (e.g., Omernik’s (1987) ecoregion map) is often used to control for natural variability during development of biological indices (e.g., Moog et al. 2004; Stoddard et al. 2008; Veselka et al. 2010; Minnesota Pollution Control Agency 2015). For wetlands, wetland vegetation type is also an important natural driver of reference expectations. We developed a classification scheme for the NWCA based on ecoregion and wetland type to help account for continental-scale differences in wetland vegetation and regional differences in wetland chemistry, hydrology, ecology, and physical habitat.

Our goal, in developing classes or groups of sites for NWCA data analysis and reporting, was to define a set of reporting groups that maximized within-class similarity in vegetation and that were ecologically and geopolitically useful for reporting assessment outcomes. The maximum number of reporting groups was constrained by the number of available probability sites. In the USEPA’s Wadeable Stream Assessment, the goal was to have at least 50 probability sites within each reporting group to provide a regional assessment with acceptably low uncertainty (Herlihy et al. 2008). Only 967 probability sites were sampled during the NWCA. Thus, the limit on the maximum number of possible reporting groups was about 20 if each group were to have a minimum of 50 probability sites for making statistically valid estimates of condition for each reporting group. Lastly, the number of reporting groups was limited by logistical concerns associated with separately setting different condition thresholds, building separate models, and reporting conditions for a large number of reporting groups. These considerations led us to attempt to identify on the order of 5–15 reporting groups across the country.

Many existing hydrological, ecological, and/or physiographic classification schemes are available for the US (e.g., Bailey 1983; Omernik 1987). We began by considering the nine national ecoregions used in the other USEPA NARS surveys of streams and lakes (Fig. 1). Those nine ecoregions were developed by Herlihy et al. (2008) by aggregating the level III ecoregional framework developed by Omernik (1987) in relation to a national ordination of stream macroinvertebrate assemblages. The complexity of wetlands, however, goes beyond just ecoregion and requires the use of both ecoregion and wetland type to create reporting groups. For wetland type, we began with the seven target wetland types (see Table 1) used in the NWCA survey design (Olsen et al. 2019; USEPA 2016b). The combination of the nine national ecoregions and the seven NWCA wetland types resulted in 56 potential groups for analysis. Further aggregation of these 56 groups was clearly necessary as most groups included fewer than 50 sampled sites and 16 groups had no sites at all. In addition to sample size constraints, we used the NWCA vegetation data to inform these aggregations because vegetation is the NWCA indicator of ecological condition.

A series of ordinations were performed using site-level data to evaluate relationships between plant species composition, NWCA wetland type, and ecoregion groupings. Ordinations based on site-level species identity and abundance (estimated as percent cover) were conducted at the national scale and by wetland type groups. Mean cover values ranged from 0 to 100 for each plant species at each site. To ensure the ordinations would reflect total species composition and not be driven by high cover dominant species, a square root transformation was applied to the data (Gauch Jr 1982; McCune and Grace 2002). Ordination results were plotted for sites based on species composition, with sites coded by symbol type to delineate wetland type, ecoregion, or ecoregion by wetland type groups. Ordinations for subsets of sites by wetland type groups were conducted using nonmetric multidimensional scaling (NMS) (R Statistical Software, version 3.1.1, “Vegan: metaMDS,” R Development Core Team 2014). The full dataset for all sampled sites was so large and complex that it was difficult to obtain a stable solution using NMS; thus, when all sites were evaluated, detrended correspondence analysis (DCA) was used for the ordinations (PC-ORD, Version 6.19, McCune and Mefford 2011). Results from all the ordinations were used to decide on the final ecoregion/wetland type composition of the NWCA reporting groups.

### Reference site screening

Because pristine conditions are uncommon or absent in most of the conterminous US, we defined *reference condition* as least disturbed (Stoddard et al. 2006). Least disturbed status for the NWCA was defined using a set of explicit quantitative criteria for specific disturbance indicators, to which all reference sites must adhere. To be designated as a least disturbed reference site, a site had to pass all of the disturbance screens or filters. This filtering process has been used previously for both regional (Waite et al. 2000) and national stream surveys (Herlihy et al. 2008), and its rationale described by Herlihy et al. (2006).

NWCA data collected in the field and laboratory were evaluated for potential utility in screening sites for disturbance (USEPA 2016b). Disturbance measures were chosen as screens based on evidence of a strong association with anthropogenic stress. Four categories of disturbance were used as screens:

- Disturbance in the buffer and AA (six indices developed)
- Hydrologic alteration in the AA (two indices developed)
- Soil chemistry in the AA (one index developed),
- Relative cover of alien plant species in the AA (one metric developed)

The ten specific screening indices used as screens are defined in Table 2.

Although water chemistry was part of the NWCA field protocol, only 56% of the wetlands sampled had sufficient surface water to collect and analyze. For this reason, and because wetland hydroperiod fluctuations—especially during the growing season when NWCA sampling occurred—can greatly influence water chemistry, water chemistry measures were excluded from the generation of the disturbance gradient.

Thresholds for each screen were set independently for each NWCA reporting group (USEPA 2016b) because the extent of human disturbance may vary greatly among ecoregions and wetland types. Initially, thresholds were set to zero human disturbance, with the exception of a 5% threshold for relative cover of alien plant species. Sites meeting these thresholds could be considered minimally disturbed reference sites (Stoddard et al. 2006). If a reporting group had a sufficient number of sites passing all these thresholds, then these zero thresholds were used to define reference sites. If there were an insufficient number of sites passed using zero thresholds, we had to relax the thresholds to obtain a sufficient number of reference sites for data analysis. Thresholds were relaxed so that approximately 15–25% of the sites in the reporting group passed the filters and these sites were used as the least disturbed reference sites for the reporting group.

All 1138 sites sampled in the NWCA were considered potential reference sites and were passed through the screening process. For the ~ 8% of sites that had repeat sample visits (for temporal variability assessment and quality assurance purposes), only the data from the first sample visit was screened and used to determine whether a site was reference or not.

### Defining the disturbance gradient

In addition to defining least disturbed reference sites, we wanted to define a disturbance gradient for NWCA sites by categorizing them into least, intermediate, or most disturbed categories. Most disturbed sites on the gradient were defined using a filtering process that paralleled the approach used for least disturbed sites. The same ten measures of disturbance (Table 2) were used and thresholds for most disturbed condition were set for each of the measures for each reporting group. If any single threshold for any measure was exceeded, the site was considered a most disturbed site. We sought to have a minimum of 15–20 most disturbed sites in each reporting group to have a sufficient sample size for stressor gradient analyses and VMMI development. As “most disturbed” is a relative definition, this lead to a goal of defining roughly 20–30% of the sites in a reporting group as most disturbed, and thresholds were set accordingly. Finally, we assigned sites not falling into either the least or most disturbed category to the intermediate disturbance category. Thus, all 1138 NWCA sites were categorized as either least, intermediate, or most disturbed.

## Results and discussion

### Defining reporting groups

**Fig. 2:**
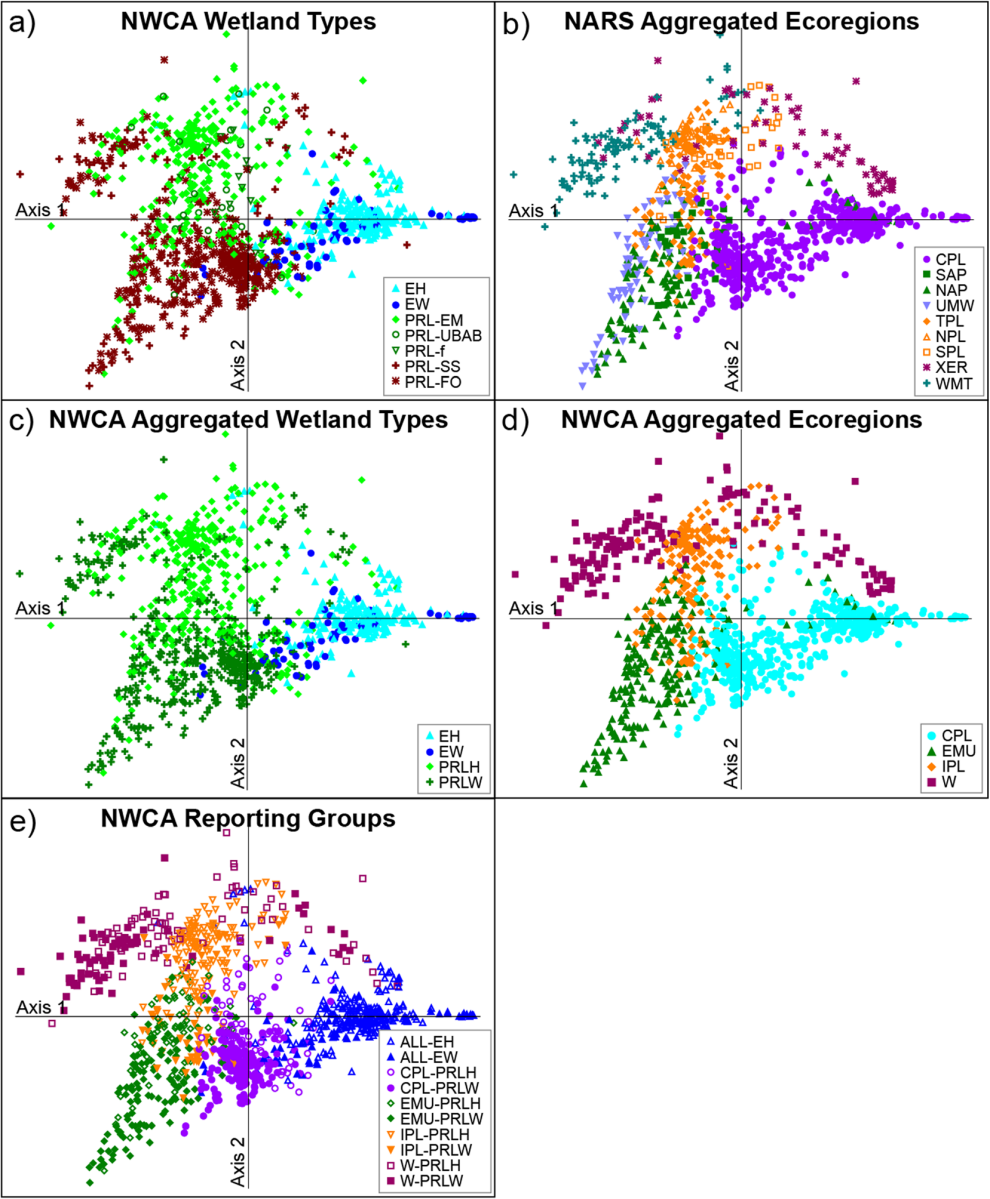
Detrended correspondence analysis ordination of NWCA sample sites based on plant species composition (presence and abundance) with sites coded by **a)** the seven NWCA wetland types, **b**) the nine aggregated NARS ecoregions, **c)** the four NWCA aggregated wetland types, **d)** the four NWCA aggregated ecoregions, and **e)** the ten NWCA ecoregion by wetland type reporting groups. Acronyms in panels **a** and **b** are defined in Table 1, and acronyms used in panels **c**–**e** are defined in Table 3

Vegetation patterns observed in the NMS ordinations representing individual wetland type groups tended to parallel those observed in the all-sites DCA ordination, so for simplicity and to provide a national overview, we present only the national-scale results, here. All 3547 observed taxa (native and nonnative) were included in the analysis. The all-sites DCA ordination was plotted five times with the sites alternatively coded to represent either the seven NWCA target wetland types (Fig. 2a), nine NARS ecoregions (Fig. 2b), four aggregated wetland types (Fig. 2c), four aggregated ecoregions (Fig. 2d), and the final NWCA reporting groups (Fig. 2e). The patterns shown by the various classifications were used to help inform the definition and selection of the final NWCA reporting groups. Ordination plots were constructed using raw site scores and unrotated axes (McCune and Mefford 2011). Eigenvalues for axes 1 and 2 were 0.908 and 0.767, respectively; with a Monte Carlo randomization test (999 permutations) having *p* = 0.0001 for both axes. The distribution of sites across the ordination is related to latitude (axis 1, Pearson *r* = − 0.666) and longitude (axis 2, Pearson *r* = − 0.698). Sites are loosely arranged along a north to south direction from left to right of the ordination diagram paralleling axis 1 and from west to east from top to bottom of the ordination diagram paralleling axis 2.

Gradient length for each DCA axis reflects standard deviations (SD) in species composition; and sites with scores that differ by more than 4 SD are expected to have no species in common (McCune and Grace 2002; Jongman et al. 1995). The gradient length for axis 1 was 13.86 and for axis 2 was 10.76. This means that from one edge of axis 1 to the other (i.e., moving from left to right across the ordination), there are more than three complete turnovers in species composition. Similarly, for axis 2 (i.e., moving from top to bottom of the ordination), there are approximately 2.5 turnovers in species composition. This level of beta diversity is not surprising given the geographic scope of the study area and the number of wetland types considered. It does, however, illustrate the need for encompassing this natural variation in defining and selecting least disturbed sites to ensure a sufficient number of reference sites to represent this diversity.

Our first step in identifying appropriate reporting groups was to evaluate patterns in species composition related to the seven wetland types (Fig. 2a, Table 1) making up the NWCA target population and to the nine NARS aggregated ecoregions (Fig. 2b, Table 1). Both of these plots show distinct to intergrading groups of sites associated with wetland type or ecoregion. For example, in Fig. 2a, estuarine wetland types were clearly separated from inland (palustrine, shallow riverine, or shallow lacustrine (PRL)) wetland types. In addition, the collective PRL woody (PRL-SS, PRL-FO) types and collective PRL herbaceous (PRL-EM, PRL-UBAB, PRL-f) types form fairly distinct groups (see Table 1 for wetland type code definitions). However, among the inland woody types, the shrub-dominated and forest-dominated wetland sites tended to intergrade somewhat across the ordination. In addition, some shrub-dominated wetland sites were interspersed among herbaceous inland wetland sites. The three types of herbaceous inland wetlands (PRL-EM, PRL-UBAB, and PRL-f) did not separate distinctly from one another. Across the estuarine group on the ordination, woody dominated (estuarine shrub/forested (EW)) systems tended to intermix at the group edges with the more abundant herbaceous (estuarine emergent (EH)) systems.

In Fig. 2b, several clear clusters of sites related to the nine NARS ecoregions were evident, especially for the Coastal Plain (CPL), Western Mountains (WMT), and the Xeric West (XER). The WMT and XER groups tend to intermingle slightly where they abut on the ordination. In other cases, sites from an individual ecoregion generally aggregate together, but intermix somewhat with geographically adjacent ecoregions. For example, sites from the Southern (SAP) and Northern (NAP) Appalachians tend to plot together into a relatively cohesive group. Sites from the Upper Midwest (UMW) plot loosely together, but intermix in places with the Appalachian group. Interspersed, on the ordination within the upper half of both the Appalachian and UMW groups are some sites from the Temperate Plains (TPL). Immediately above this area of mixing and between the WMT and XER groups, sites representing all three interior plains ecoregions (TPL, Northern (NPL), and Southern (SPL)) group together.

The observed patterns of species composition make sense ecologically. We expected sites from different ecoregion or wetland type groups to form fairly distinct clusters on the ordination. However, we also anticipated there would be some level of intergrading at the edges of these clusters where sites were relatively closer together on the landscape or were from similar wetland types. In addition, it is likely that the nonnative species with wide ecologic amplitude and widespread distribution will have had some level of homogenizing influence on the species composition (Magee et al. 2008), resulting in decreasing distinctness between groups on the ordination. Not surprisingly, there also appears to be an interaction between wetland type (Fig. 2a) and ecoregion (Fig. 2b). For example, looking at the separation of wetland types (Fig. 2a) for the set of sites representing the WMT ecoregion (Fig. 2b), the inland woody sites (PRL-SS, PRL-FO) tend to separate from the inland herbaceous sites (primarily PRL-EM in this ecoregion). Within the XER sites (Fig. 2b), the inland herbaceous (PRL-EM), inland woody (PRL-SS, PRL-FO), and the estuarine emergent (EH) sites generally separate from one another (Fig. 2a). Across the CPL (Fig. 2b), the estuarine and inland wetland types separate from each other, and within each of these two groups herbaceous and woody types tend to separate (Fig. 2a). The eastern mountains (SAP, NAP) and Upper Midwest (UMW) (Fig. 2b) tend to be dominated by inland woody wetland types, whereas the interior plains ecoregions (TPL, NPL, SPL) inland herbaceous types are more common (Fig. 2a).

**Fig. 3:**
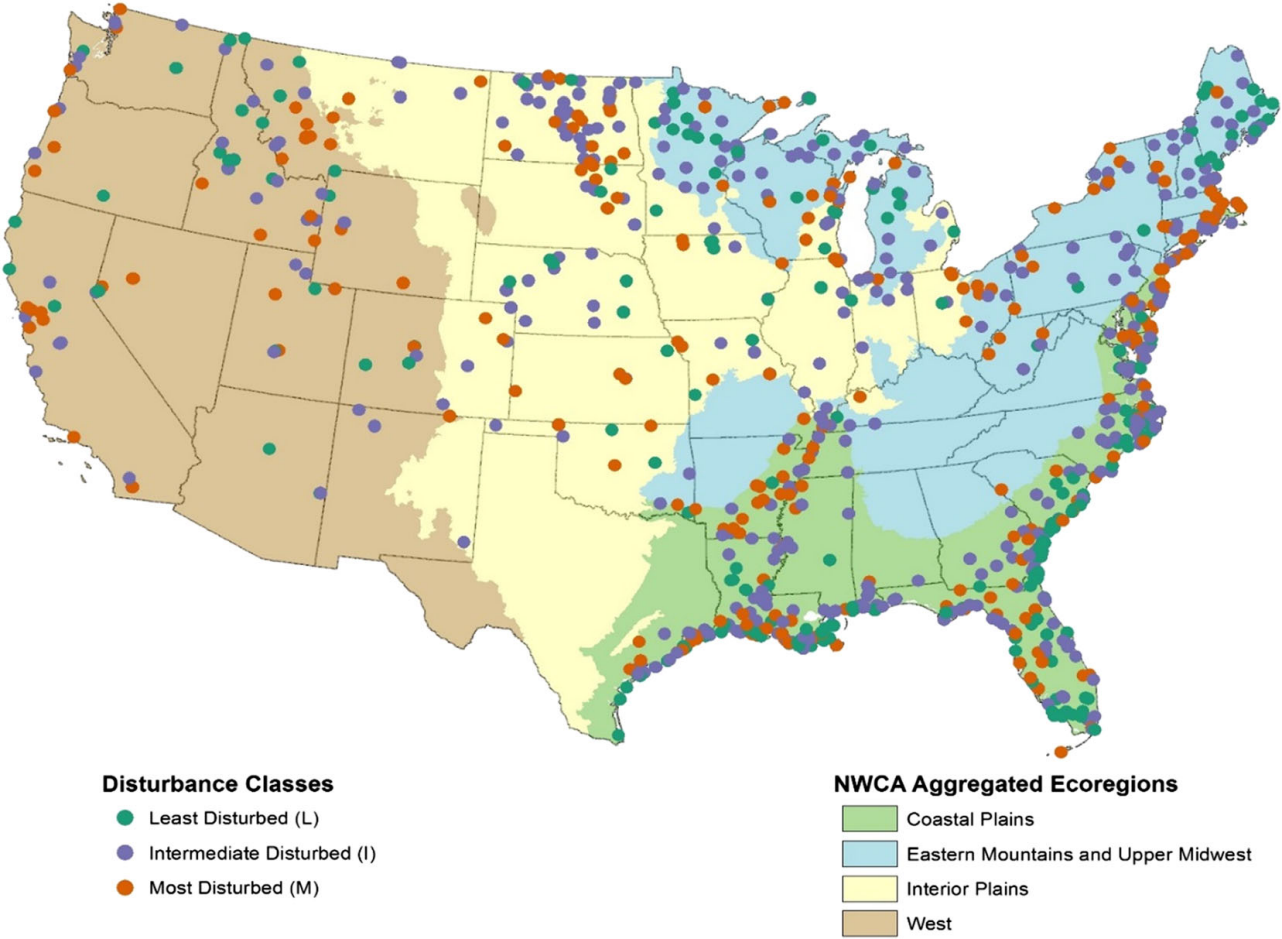
Map of the conterminous US showing the four NWCA aggregated ecoregions and the location of the NWCA sites coded by disturbance class (least, intermediate, or most disturbed)

Our goal was to identify site groups, which minimize within-group differences in species composition and limit overlap between groups, within the constraints of maintaining minimum sample sizes for individual groups. Sample size constraints required consolidation of the 56 potential groups that could be formed by the intersection of the seven NWCA wetland types and the nine NARS ecoregions. Based on the vegetation patterns from the ordination plots in Fig. 2a, b, we aggregated the seven NWCA wetland types into four NWCA aggregated wetland types (Fig. 2c, Table 3, top row) and the nine NARS national ecoregions into four NWCA aggregated ecoregions (Figs. 2d and 3, Table 3, left-most column). The aggregated wetland types (Fig. 2c) and aggregated ecoregions (Fig. 2d) form fairly distinct groups based on species composition, with individual groups tending to intergrade somewhat where they abut on the ordination.

**Table 3:** Matrix showing the four NWCA aggregated ecoregions (left-most column) and the four NWCA aggregated wetland types (top row) used to form the 10 NWCA reporting groups by intersecting ecoregion and wetland type. Note estuarine reporting groups are formed nationally (ALL) and not by ecoregion due to sample size limitations. Acronyms for all groups are in parentheses following their names as well as the total number of NWCA sites in each reporting group

NWCA aggregated ecoregion^a^	Palustrine, Riverine, and Lacustrine Herbaceous (PRLH)	Palustrine, Riverine, and Lacustrine Woody (PRLW)	Estuarine Herbaceous (EH)	Estuarine Woody (EW)
Coastal Plains (CPL)	Coastal Plains Herbaceous (CPL-PRLH) *n* = 72	Coastal Plains Woody (CPL-PRLW) *n* = 189		
Eastern Mountains and Upper Midwest (EMU)	Eastern Mountains and Upper Midwest Herbaceous (EMU-PRLH) *n* = 73	Eastern Mountains and Upper Midwest Woody (EMU-PRLW) *n* = 127		
Interior Plains (IPL)	Interior Plains Herbaceous (IPL-PRLH) *n* = 138	Interior Plains Woody (IPL-PRLW) *n* = 52		
West (W)	West Herbaceous (W-PRLH) *n* = 67	West Woody (W-PRLW) *n* = 75		
National (ALL)			Estuarine Herbaceous (ALL-EH) *n* = 272	Estuarine Woody (ALL-EW) *n* = 73

Aggregates of NWCA wetland types (see Table 1 for acronyms); PRLH = PRL-EM + PRL-f + PRL-UBAB, PRLW = PRL-FO + PRL-SS, EH = EH, EW = EW

^a^Aggregates of NARS nine national ecoregions (see Table 1 for acronyms); CPL = CPL, EMU = NAP + SAP + UMW, IPL = TPL + SPL + NPL, and W = XER + WMT

To address the interaction between ecoregion and wetland type, the four aggregated ecoregions and four wetland types were combined to develop 10 reporting groups (Fig. 2e, Table 3) for the NWCA. For each of the NWCA ecoregions, separate reporting groups were defined for the inland herbaceous (PRLH) and the inland woody (PRLW) wetland types, giving eight reporting groups. Most estuarine sites sampled in the NWCA occurred in the CPL, only a small number were in the EMU or W, and none occurred in the IPL. Consequently, the estuarine wetlands were not separated by ecoregion and only two estuarine reporting groups were defined, one for herbaceous systems (EH) and one for woody systems (EW). It is important to remember the large scale of the reporting groups and the length of the DCA axis gradients in species composition. Despite the cohesiveness of the groups on the ordination plot, there may still be substantial turnover in species composition from edge to edge across any particular group. This variation may suggest that effective vegetation metrics for distinguishing sites with least disturbed vs. most disturbed condition will be metrics that are applicable across broad vegetation types (Magee et al. 2019a, USEPA 2016b). Nevertheless, the resulting NWCA regionalization provided reporting groups with sufficient sample sizes (Table 3) for within-group analyses to support disturbance indicator development, reference site selection, and development of the disturbance gradient.

Partitioning the effects of natural factors from the effects of anthropogenic stressors is a critical component of nearly all bioassessment programs and is necessary for obtaining the greatest accuracy and precision in the specification of the reference condition for each assessed site (Herlihy et al. 2008). The intersecting large-scale ecoregions and wetland types used to define reporting groups were useful in accounting for variation in vegetation at a continental scale (shown by the DCA ordination; Fig. 2). The NWCA reporting groups not only minimize within-group variation in species composition, but can also be expected to provide ecological partitioning that is appropriate for characterizing physical attributes of the natural environment in each group. Setting reference condition within each reporting group allows separation, at least in part, of the characterization of human disturbance from the natural biotic and environmental variation present for each group. The reporting groups in Table 3 were used throughout data analysis and reporting in the NWCA for (1) defining reference condition and the disturbance gradient (this paper), (2) developing and calibrating the VMMI and determining VMMI condition class thresholds (Magee et al. 2019a), and (3) evaluating the extent of stressors (Lomnicky et al. 2019) and their potential impact (Herlihy et al. 2019).

### Identifying least disturbed reference sites

Thresholds distinguishing least disturbed sites were set independently for all ten NWCA reporting groups (Table 4) as the extent of human disturbance can vary greatly among ecoregions and wetland types. Initially, thresholds were set to zero human disturbance for each disturbance index, with the exception of alien plant species cover where the threshold was set to 5%. These thresholds became the definition of a minimally disturbed reference site (Stoddard et al. 2006). In four reporting groups (ALL-EW, ALL-EH, EMU-PRLW, CPL-PRLW), a sufficient number of reference sites could be defined using screening thresholds for the minimally disturbed definition (Table 4). In the other six reporting groups, we had to relax the thresholds to obtain a sufficient number of least disturbed reference sites for data analysis (Table 4). Most groups only required limited threshold relaxation to achieve a sufficient number of sites but the W-PRLH group required a relatively high degree of threshold relaxation to 20% relative cover of alien plant species and to a summary buffer disturbance index of > 1.2. Industrial disturbance was rarely observed in the NWCA so the B1H_IND screening threshold was always zero.

**Table 4:** Disturbance measure threshold values for sites to be categorized as least disturbed by reporting group. If any single threshold was exceeded at a site, the site was *not* considered least disturbed. An index score of 0 indicates disturbance not present. See Table 3 for definitions of reporting group acronyms

Reporting group	B1H_AGR (agriculture)	B1H_RESURB (residential/urban)	B1H_HYD (hydrology)	B1H_IND (industry)	B1H_HAB (habitat)	B1H_ALL (summary)
ALL-EW	> 0	> 0	> 0	> 0	> 0	> 0
ALL-EH	> 0	> 0	> 0	> 0	> 0	> 0
EMU-PRLW	> 0	> 0	> 0	> 0	> 0	> 0
EMU-PRLH	> 0	> 0.10	> 0	> 0	> 0.10	> 0.10
CPL-PRLW	> 0	> 0	> 0	> 0	> 0	> 0
CPL-PRLH	> 0	> 0	> 0	> 0	> 0.20	> 0.20
IPL-PRLW	> 0.10	> 0.10	> 0.10	> 0	> 0.20	> 0.20
IPL-PRLH	> 0.15	> 0.15	> 0.15	> 0	> 0.15	> 0.30
W-PRLW	> 0.10	> 0.10	> 0.10	> 0	> 0.10	> 0.10
W-PRLH	> 0.60	> 0.60	> 0.60	> 0	> 1.00	> 1.20
Reporting group	Hydrology high impact	Hydrology moderate impact	Soil chemistry heavy metal index		Relative cover of alien plant species	
ALL-EW	> 0	> 0	> 0		> 5%	
ALL-EH	> 0	> 0	> 0		> 5%	
EMU-PRLW	> 0	> 0	> 0		> 5%	
EMU-PRLH	> 0	> 0	> 1		> 5%	
CPL-PRLW	> 0	> 0	> 0		> 5%	
CPL-PRLH	> 0	> 1	> 0		> 5%	
IPL-PRLW	> 0	> 1	> 2		> 5%	
IPL-PRLH	> 1	> 1	> 2		> 20%	
W-PRLW	> 0	> 1	> 2		> 5%	
W-PRLH	> 1	> 1	> 1		> 20%	

Of the 1138 NWCA sites, 277 (24%) were determined to be least disturbed reference sites (Table 5). The number of least disturbed reference sites varied by region from 12 in IPL-PRLW to 100 (or 37%) in ALL-EH. With the exception of the industrial buffer screen which eliminated very few sites from least disturbed status, the other nine disturbance screening criteria eliminated similar proportions of sites (Table 6). The habitat buffer screen eliminated the highest percentage (37%) of sites from least disturbed status followed by high hydrologic disturbance (27%), alien plant cover (24%), and hydrologic modifications in the buffer (24%). The percentages in Table 6 add up to well over 100% because it was common for a site to be eliminated by screens for multiple disturbance measures.

**Table 5:** Sample site distribution of least disturbed and most disturbed sites by reporting groups. See Table 3 for the definition of reporting group acronyms

Reporting group	Total number of sites screened	Number of least disturbed sites	Percent least disturbed sites	Number of most disturbed sites	Percent most disturbed sites
ALL-EW	73	16	22%	19	26%
ALL-EH	272	100	37%	82	30%
EMU-PRLW	127	21	17%	27	21%
EMU-PRLH	73	16	22%	24	33%
CPL-PRLW	189	37	20%	55	29%
CPL-PRLH	72	16	22%	20	28%
IPL-PRLW	52	12	23%	14	27%
IPL-PRLH	138	26	19%	42	30%
W-PRLW	67	16	24%	21	31%
W-PRLH	75	17	23%	27	36%
All NWCA Sites	1138	277	24%	331	29%

**Table 6:** Sensitivity of the different screening criteria used to define the disturbance gradient. The “not least disturbed” column relates the percent of all NWCA sites that exceeded the least disturbed criteria for that particular screen. The “most disturbed” column relates the percent of all NWCA sites that exceeded the most disturbed criteria for that particular screen

Screen	% sites not least disturbed	% sites most disturbed
Buffer–agriculture	22	3.5
Buffer–residential/urban	16	1.7
Buffer–hydrology	24	4.6
Buffer–industrial	0.9	0.1
Buffer–habitat	37	6.9
Hydrology–high disturbance	27	12
Hydrology–medium disturbance	11	0.7
Soil chemistry–heavy metals	18	3.3
Relative cover of alien plant species	24	5.3

Use of reference site data is a fundamental requirement for most bioassessment surveys. The extensive nature of anthropogenic disturbance, however, has often made finding reference sites an extremely difficult process. The difficulty tends to increase with the scale of the survey. In more localized surveys, it is possible to census or intensively examine a large proportion of the study population for reference suitability. For example, in southeastern Arkansas, Justus (2010) looked at all lakes with available water quality data followed by field reconnaissance and intensive sampling to identify a reference lake as the one with the least impairment in each of their four lake classes. As the scale of study increases and becomes continent-wide, this level of intensive effort is not practical. In addition, sites tend to be selected with a large element of professional judgment resulting in a definition of reference that is difficult, if not impossible, to replicate and which is qualitative instead of quantitative.

Although we were able to gather landscape data (e.g., land use within a 1-km buffer of the AA) using GIS layers, we opted not to use these data as additional screening criteria for reference status. We made this decision for a number of reasons. For one, GIS layers reflect conditions present at the time of digital data acquisition rather than at the time of field sampling and provide less specificity compared to the data gathered in the field. In addition, we wanted to include the possibility that wetlands in good condition exist in what is considered an “impacted” landscape. Also, land use activity in a predefined buffer of fixed size may not reflect conditions in the study site, especially with respect to intensity of disturbance. We also wanted to avoid labelling landscape-level disturbances such as agriculture as “bad.” What really matters is whether the effects of the disturbance are reaching the study site, and this was better reflected by on the ground field data than remote landscape data. Therefore, we only used information directly measured by field crews, on the ground, to establish the disturbance gradient and classify reference sites.

### Minimal versus least disturbed reference condition

Because pristine conditions are uncommon or absent in many places across the continent, or even globally, the 2011 NWCA followed the practice of previous NARS assessments and defined reference condition as least disturbed (Paulsen et al. 2008; USEPA 2009). Least disturbed is defined as those sites with the best available physical, chemical, and biological condition given the current status of the landscape in which the site is located (Stoddard et al. 2006). Examination of the NWCA least disturbed sites designated in our screening process revealed that a number of the sites also met the definition of minimally disturbed. Minimally disturbed sites were identified by setting the thresholds for the ten NWCA disturbance indices to zero, i.e., indicating that none of the indicators of disturbance considered in Table 2 were present in the AA and buffer of the sites being screened. Of the original 277 least disturbed sites (Table 5), 170 were minimally disturbed with the vast majority of these located in the estuarine reporting groups.

NWCA wetland reference sites in the EMU-PRLW, the Coastal Plain reporting groups, and the Estuarine reporting groups were generally categorized as minimally disturbed by our data. Over half the reference sites in each of these five reporting groups had zero human disturbance scores for the ten NWCA screening filters. On the other hand, reference wetlands in the West, Interior Plains PRL herbaceous and woody reporting groups, and in the PRL herbaceous reporting group in the Eastern Mountains and Upper Midwest, should largely be considered least disturbed. We had to relax the screening thresholds and accept some wetlands with increasing levels of disturbance to find sufficient reference wetlands in those areas. The largest relaxation of the screening thresholds was necessary in the W-PRLH where no minimally disturbed sites were found. The least disturbed concept has also been used to identify reference streams by Yates and Bailey (2010) in Ontario and by Baattrup-Pedersen et al. (2009) in Denmark. In Denmark, they found that upon examination, none of the 128 a priori selected reference streams fulfilled all reference criteria and only 3 passed when the criteria were less strict. However, they did not recommend relaxing criteria but concluded that there is a need for alternative methods to establish reference condition in Danish streams.

The variation between least disturbed and minimally disturbed reference condition (Stoddard et al. 2006) among the different reporting groups has important implications for bioassessment. Many assessment methods depend on a population of reference sites for statistical modeling and setting thresholds. In the NWCA, reference sites were used in developing the VMMI and setting the good/fair/poor condition class thresholds for both the VMMI (Magee et al. 2019a) and soil phosphorus (USEPA 2016b). Varying reference site quality among the different reporting groups means that assessment results may not be directly comparable between groups. Groups with poorer quality reference sites may have a lower bar for defining good condition than higher quality reference site groups. This has historically been a very difficult problem for any large-scale assessment driven by the fact that minimally disturbed reference sites are rare or absent in many parts of the world.

### Identifying most disturbed sites

Most disturbed sites were defined using a filtering process paralleling that for least disturbed sites. Our objective was to define approximately 20–30% of the sites in a reporting group as most disturbed and thresholds were set accordingly (Table 7). As a result, reporting group screening threshold values for defining most disturbed condition varied among reporting groups. Most disturbed threshold values followed the same general pattern as was observed for defining the threshold values for the least disturbed sites. The Estuarine, EMU-PRLW, and CPL-PRLW reporting groups had the lowest threshold values, while the W-PRLH group had the highest. The most disturbed screening threshold for the overall buffer index in the W-PRLH was > 2.0 (Table 7). Alien plant cover > 50% was used as a most disturbed threshold in all reporting groups.

**Table 7:** Disturbance threshold values for sites categorized as most disturbed by reporting group. If any single threshold was exceeded at a site, it was considered most disturbed. See Table 3 for definitions of reporting group acronyms

Reporting group	B1H_AGR (agriculture)	B1H_RESURB (residential/urban)	B1H_HYD (hydrology)	B1H_IND (industry)	B1H_HAB (habitat)	B1H_ALL (summary)
ALL-EW	> 0.25	> 0.25	> 0.25	> 0.25	> 0.25	> 0.75
ALL-EH	> 0.25	> 0.25	> 0.25	> 0.25	> 0.25	> 0.75
EMU-PRLW	> 0.25	> 0.25	> 0.25	> 0.25	> 0.50	> 1.00
EMU-PRLH	> 0.30	> 0.30	> 0.30	> 0.30	> 0.60	> 1.00
CPL-PRLW	> 0.25	> 0.25	> 0.25	> 0.25	> 0.50	> 1.00
CPL-PRLH	> 0.60	> 0.60	> 0.60	> 0.60	> 1.00	> 1.50
IPL-PRLW	> 0.30	> 0.30	> 0.30	> 0.30	> 0.60	> 1.00
IPL-PRLH	> 0.60	> 0.60	> 0.60	> 0.60	> 1.20	> 1.80
W-PRLW	> 0.60	> 0.60	> 0.60	> 0.60	> 0.80	> 1.00
W-PRLH	> 0.75	> 0.75	> 0.75	> 0.75	> 1.50	> 2.00
Reporting group	Hydrology high impact	Hydrology moderate impact	Soil chemistry heavy metal index		Relative cover of alien plant species	
ALL-EW	> 1	> 1	> 2		> 50%	
ALL-EH	> 1	> 1	> 2		> 50%	
EMU-PRLW	> 1	> 1	> 2		> 50%	
EMU-PRLH	> 2	> 2	> 2		> 50%	
CPL-PRLW	> 1	> 1	> 2		> 50%	
CPL-PRLH	> 2	> 2	> 2		> 50%	
IPL-PRLW	> 1	> 2	> 2		> 50%	
IPL-PRLH	> 1	> 2	> 2		> 50%	
W-PRLW	> 2	> 2	> 3		> 50%	
W-PRLH	> 3	> 3	> 3		> 50%	

Of the 1138 NWCA sites, 331 (29%) were categorized as most disturbed (Table 5). The percentage of sites that were most disturbed in each reporting group ranged from 21% in EMU-PRLW to 36% in W-PRLH. As shown in Table 6, the habitat buffer screen placed the highest percentage of sites (6.9%) into most disturbed status followed by alien plant cover (5.3%) and hydrologic modifications in the buffer (4.6%).

### Defining the disturbance gradient

We classified the sites not falling into either least or most disturbed categories into an intermediate disturbance category to finalize the disturbance gradient for all NWCA sites. In general, the distribution of least disturbed reference sites and most disturbed sites are spread out reasonably well across the NWCA sample (Fig. 3). Looking at general patterns in distribution of sites among disturbance categories for the NWCA wetland types (Table 8), it is not surprising that least disturbed sites were uncommon for previously farmed PRL (palustrine, shallow riverine, or shallow lacustrine) wetlands (PRL-f). In contrast, estuarine herbaceous (EH) and PRL-unconsolidated bottom/aquatic bed (PRL-UBAB) types tended to have a greater percentage of least disturbed sites than other NWCA wetland types. The distribution of least and most disturbed sites across HGM classes (Table 8) was similar among the classes. Tidal and fringe wetlands tended to be a bit less disturbed than the other classes as was seen with the estuarine wetland types.

**Table 8:** Percent of the 1138 sites screened in each disturbance category by NWCA wetland type and hydrogeomorphic class (Brinson 1993). Numbers are rounded and may not add to 100%

Wetland type	% least disturbed sites	% intermediate disturbed sites	% most disturbed sites
NWCA wetland type			
PRL-emergent (PRL-EM)	21	48	32
PRL-unconsolidated bottom/aquatic bed (PRL-UBAB)	38	38	23
PRL-farmed (PRL-f); subset not actively farmed	5	55	41
PRL-shrub/scrub (PRL-SS)	14	53	33
PRL-forested (PRL-FO)	23	54	23
Estuarine emergent (EH) [estuarine herbaceous]	37	33	30
Estuarine shrub/forest (EW) [estuarine woody]	22	52	26
Hydrogeomorphic class (HGM)			
HGM-depression	13	55	31
HGM-flats	24	48	28
HGM-fringe	41	41	18
HGM-riverine	20	51	28
HGM-slope	24	43	33
HGM-tidal	35	35	30

*PRL* palustrine, shallow riverine, or shallow lacustrine wetlands

**Fig. 4:**
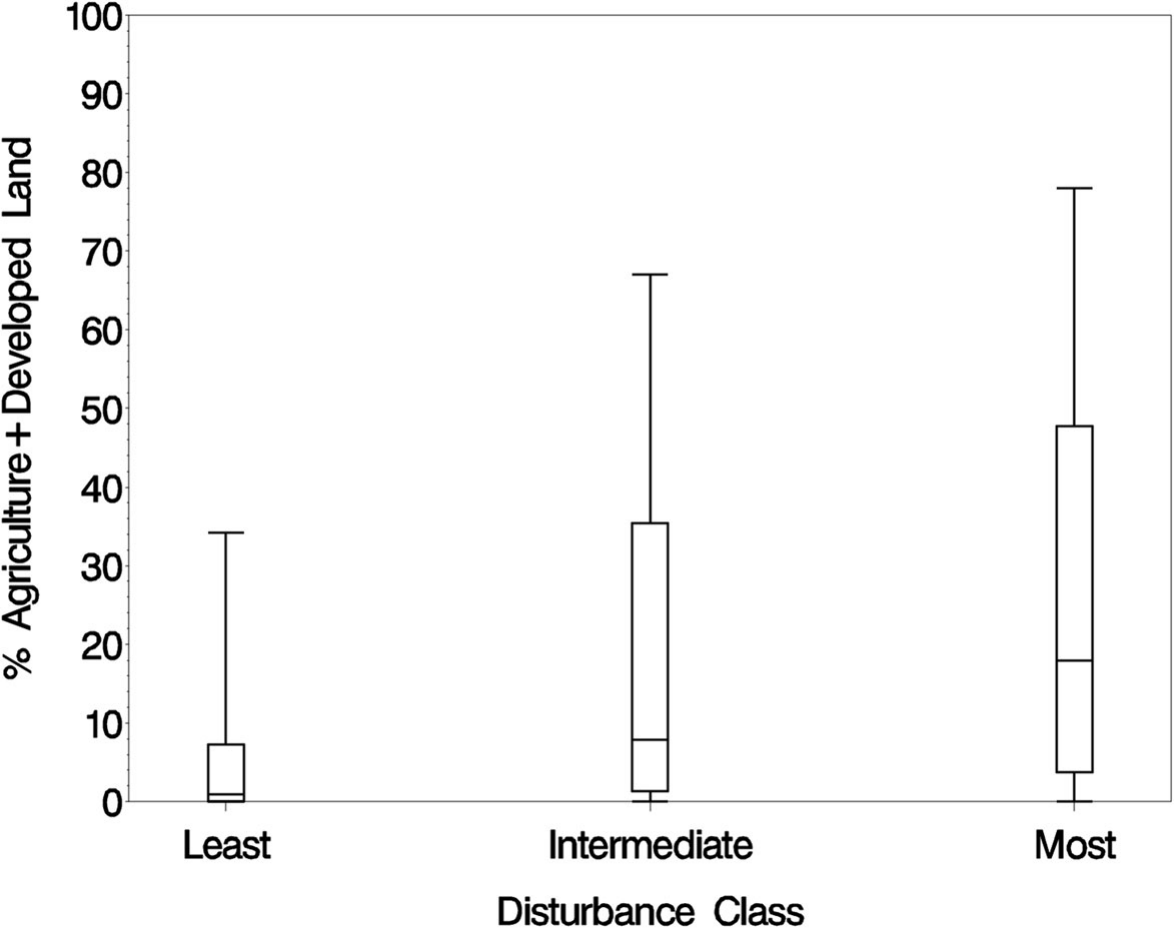
Box and whisker plots of percent agriculture and developed land in a 1-km radius circle around each sample point by disturbance class. Boxes show the median and interquartile range; whiskers show the 10th/90th percentiles

We evaluated the relationship of the three disturbance categories to GIS land cover data using box and whisker plots; plotting the percent cover of developed land and land in agriculture use within a 1-km circular buffer around each site (Fig. 4). By one-way ANOVA, there was a highly significant disturbance category effect (*F* = 44.7, *p* < 0.0001) with all three categories significantly different from one another (*p* < 0.0001). Median percent disturbed land was 0.9% in least disturbed sites, 7.8% in intermediate sites, and 18% in most disturbed sites. The percentage of sites with zero disturbed land decreased by category from 41% in least disturbed to 18% in intermediate to 12% in most disturbed.

The list of screening criteria in Table 2 that we used to define the disturbance gradient cannot be considered a complete list of all factors influencing reference status. Ideally, a more inclusive set of disturbance factors would be used to identify reference sites. However, many disturbances cannot be observed in a one-time site visit, nor from landscape data. For example, direct information on the temporal dynamics of hydrologic alteration, the input of organic contaminants, or historical landscape changes were not available. In this sense, our screening approach probably allowed some sites affected by important anthropogenic stressors to pass through the screening process and be identified as least disturbed. Nevertheless, the 10 site-level disturbance factors used in screening produced a broad characterization of the physical/chemical environment at a site. With the exception of industrial disturbance (which was very rare), each of the other nine disturbance filter screens identified more than 10% of the NWCA sites as not least disturbed (Table 6).

It is difficult to address the adequacy of a set of reference sites for a national assessment like the NWCA. Ode et al. (2016) evaluated the suitability of their reference streams in California against two performance criteria, success in rejecting sites with poor biology in least-stressed relative to most-stressed sites, and the representativeness of the pool of reference sites relative to the natural gradients in the population. In the NWCA, the VMMI was the only assessed biological metric done at all sites, but it was built using the reference site data, so it would be circular reasoning to use the VMMI to address reference site quality. We lacked other biological data to test the disturbance gradient but we did test the gradient against GIS landscape-level stressor land cover data which showed only limited agricultural and developed land in the 1-km buffer around least disturbed reference sites (Fig. 4). Over half the reference sites have < 1% agriculture and developed land cover in their buffer. Being a national randomized survey, the NWCA has a wide mix of wetland types and hydrologic settings, so it is difficult to test or even define representativeness. We did examine reference site representativeness by looking at spatial plots of least versus most disturbed site locations and the distribution of the disturbance gradient across HGM classes. Reference sites seemed spatially representative; they were spread out across the country (Fig. 3), helped by the fact that we picked reference sites for each of the ten reporting groups. One of the main reasons to pick reference sites by reporting groups was to insure representativeness across the broad ecoregion and vegetation type classes represented by the reporting groups. Reference sites were also well distributed among all the HGM classes so they were not all one HGM type (Table 8).

### Evaluating the disturbance gradient in handpicked sites

The 150 handpicked wetland sites were selected by NWCA partners as potential reference sites. The disturbance level observed at these handpicked sites was evaluated along with all sampled NWCA sites using the same quantitative screening thresholds (Tables 4 and 7). Of the 150 handpicked sites, 72 (48%) were identified as least disturbed, 68 (45%) were intermediate, and 10 (7%) were most disturbed (Table 9). The percentage of handpicked sites that passed screening criteria for least disturbed status varied widely among reporting groups. In the W-PRLH and CPL-PRLH, over 90% of the handpicked sites were least disturbed compared to less than 40% of the handpicked sites in the EMU-PRLW, CPL-PRLW, and IPL-PRLW. Overall, handpicked woody sites were less likely to have been categorized as least disturbed than herbaceous handpicked sites.

**Table 9:** Number (and percent) of the 150 handpicked sites assigned to disturbance category in each NWCA reporting group based on the NWCA quantitative disturbance screens

Class wetland type	Least disturbed sites	Intermediate disturbed sites	Most disturbed sites
ALL-EW	2 (50%)	1 (25%)	1 (25%)
ALL-EH	10 (71%)	4 (29%)	0
EMU-PRLW	14 (33%)	27 (63%)	2 (5%)
EMU-PRLH	7 (41%)	9 (53%)	1 (6%)
CPL-PRLW	10 (38%)	15 (58%)	1 (4%)
CPL-PRLH	9 (90%)	1 (10%)	0
IPL-PRLW	3 (38%)	3 (38%)	2 (25%)
IPL-PRLH	12 (57%)	7 (33%)	2 10%)
W-PRLW	2 (50%)	1 (25%)	1 (25%)
W-PRLH	3 (100%)	0	0
Total	72 (48%)	68 (45%)	10 (7%)

Candidate reference sites are often identified based on BPJ. This may be less problematic in smaller surveys where all judgments are made by 1 person or a single team and are therefore more internally consistent. However, in surveys over large regions, BPJ often involves combining lists of potential reference sites from many people. Reference condition means different things to different people in different places and may vary with study objectives; thus, BPJ lists can contain unknown biases and inconsistent definitions of reference. Our experience with past national surveys is that a large proportion of BPJ-selected reference sites turn out to be non-reference when analyzed in a consistent manner based on field data (Herlihy et al. 2008, 2013). Similar results were found in the NWCA, despite a significant amount of effort spent in office screening candidate reference sites. Of the 150 handpicked candidate sites, 78 (52%) turned out to be non-reference when screened using the disturbance indicator thresholds in Table 4. There was no consistent spatial pattern to the failure rate in selecting reference sites, and it varied widely among the different reporting groups, ranging from 0 to 68% (Table 9).

### Summary and conclusions

NWCA vegetation data were used to inform the definition of 10 reporting groups based on ecoregion and wetland type that minimized the naturally occurring variation in wetland vegetation associated with continent-wide differences in biogeography. Least disturbed reference sites were identified by filtering NWCA sample data for disturbance, by reporting group, using a series of variables that included indicators of human disturbance, hydrologic alternation, soil heavy metals, and alien plant species. Defining reference condition using a set of quantitative indicators and thresholds removes much of the BPJ element from the definition of reference. It also has the advantage of adding consistency, objectivity, and reproducibility to the reference site selection process. An effort to handpick reference sites ahead of time in the NWCA had a 52% failure rate when evaluated against the defined reference criteria despite a significant effort made in pre-sample screening of candidate sites.

Our work defined a disturbance gradient for use in evaluating wetland condition across the conterminous US. Ultimately, 277 least disturbed reference sites were identified and used to set reference expectations for the NWCA. Reference sites in the EMU-PRLW, and both Coastal Plain and Estuarine reporting groups were found to generally be minimally disturbed based on our disturbance criteria. Over half the reference sites in each of these five reporting groups had zero human disturbance scores for the ten screening filters. On the other hand, our reference wetlands in both West and Interior Plains reporting groups and the EMU-PRLH must largely be considered least disturbed. We had to relax the screening thresholds and accept some wetlands with greater levels of disturbance to find sufficient reference wetlands in those areas. The NWCA provided a unique opportunity to improve our conceptual and technical understanding of how to best apply a reference condition approach to assessing wetlands across the US. These results will enhance the technical quality of future national assessments.
